# Bemiparin versus enoxaparin as thromboprophylaxis following vaginal and abdominal deliveries: a prospective clinical trial

**DOI:** 10.1186/s12884-015-0515-2

**Published:** 2015-03-28

**Authors:** Shahla K Alalaf, Rojan K Jawad, Parez R Muhammad, Mahabad S Ali, Namir G Al Tawil

**Affiliations:** Department of Obstetrics and Gynaecology, College of Medicine, Hawler Medical University, Kurdistan Region, Erbil, Iraq; Hawler Ministry of Health, Directorate of Health, Kurdistan Region, Erbil, Iraq; Department of Community Medicine, College of Medicine, Hawler Medical University, Kurdistan Region, Erbil, Iraq

**Keywords:** Bemiparin, Enoxaparin, Low-molecular-weight heparin, Postpartum thromboprophylaxis

## Abstract

**Background:**

Venous thromboembolism (VTE) is a leading cause of maternal mortality and morbidity, with the highest incidence occurring during the postpartum period. This study compared the ability of two types of low-molecular-weight heparin, enoxaparin and bemiparin, to decrease the incidence of VTE following elective caesarean section, emergency caesarean section, and vaginal delivery in women who had risk factors for thromboembolism.

**Methods:**

In this prospective clinical trial using a sequential group allocation method, 7020 haemodynamically stable women delivered vaginally or abdominally at the Maternity Teaching Hospital, Kurdistan region, Erbil, Iraq, between May 1, 2012, and November 1, 2013. These women had risk factors for VTE and were allocated to the following groups: treatment with 3500 IU/day of bemiparin, 4000 IU/day of enoxaparin, or no intervention (control). The first dose was administered 6 hours after vaginal or abdominal delivery, or 8 hours after delivery in women receiving spinal anaesthesia. Subsequent doses were administered daily for up to 6 days. The incidence of VTE was assessed for up to 40 days postpartum. Data were analyzed using the Statistical Package for Social Sciences version 19. Proportions were compared using the chi square test of association or Fisher’s exact test. Binary logistic regression analysis was used with VTE as the dependent variable.

**Results:**

VTE occurred in 1 (0.042%) woman in the bemiparin group, two (0.085%) women in the enoxaparin group, and nine (0.384%) women in the control group (P = 0.017). Regression analysis showed that women on bemiparin (OR = 0.106; 95% CI = 0.013–0.838) and enoxaparin (OR = 0.226; 95% CI = 0.049–1.049) were at lower risk of developing VTE than control women. Adverse events in the enoxaparin group included wound dehiscence, haematoma, and separation. None of these occurred in the bemiparin group.

**Conclusions:**

Postpartum bemiparin is significantly effective as a prophylaxis for VTE. Wound complications develop after use of enoxaparin, but not after bemiparin.

**Trial registration:**

ClinicalTrials.gov; Identifier: NCT01588171; date: April 26, 2012.

## Background

Venous thromboembolism (VTE) occurs in frequently. However, VTE is a leading cause of sickness and death during pregnancy and puerperium, and its diagnosis and therapy remain challenging [1]. Approximately 50% of pregnancy-related pulmonary emboli and more than 30% of pregnancy-related VTEs occur after delivery [2]. VTE during pregnancy and postpartum remains a major, but potentially preventable, cause of maternal death and morbidity. Prevention of VTE mainly involves using anticoagulants for thromboprophylaxis [3].

Evidence available from randomized clinical trials is inadequate in guiding clinical decision-making on anticoagulants during pregnancy and the postpartum period. Recommendations in guidelines have been based on case series, extrapolations from non-pregnant patients, and the opinion of experts [4]. Indeed, a recent systematic review showed insufficient evidence on which to base recommendations for prophylaxis of VTE because of poor methodology and/or small sample sizes for the research that was available [5]. Recent guidance from the Royal College of Obstetricians and Gynaecologists (RCOG) and the National Institute for Health and Clinical Excellence encourages the use of low-molecular-weight heparin (LMWH) thromboprophylaxis during pregnancy and after caesarean and vaginal deliveries for women with risk factors for VTE [6,7]. The recommendations largely depend on expert opinion and pay insufficient attention to the potential side effects of thromboprophylaxis. Additionally, previous studies on VTE did not include women who were delivered vaginally and almost all of them depended on caesarean section (CS) as a risk factor.

Among the types of LMWH that are currently used for VTE prophylaxis, bemiparin, a second-generation LMWH, and enoxaparin, a first-generation LMWH. These two LMWHs have different ratios of anti-Xa to anti-IIa activity (9.7 and 3.9, respectively) [8]. No previous studies have compared these two different LMWHs as thromboprophylaxis. The purpose of this trial was to determine the ability of bemiparin and enoxaparin, relative to no intervention, to reduce the incidence of postpartum VTE in women at risk of VTE. This trial also aimed to compare the incidence of adverse events in the two interventional groups.

## Methods

A prospective clinical trial with the sequential group allocation method was performed. We included women aged ≥15 years with risk factors for VTE who delivered vaginally or by emergency or elective CS at the Maternity Teaching Hospital, Kurdistan Region, Erbil City, Iraq, between May 1, 2012, and November 1, 2013.

VTE risk factors after vaginal and abdominal deliveries were determined based on the RCOG 2009Green-top Guideline. Women who delivered vaginally were included in the study if they had two or more persistent risk factors for VTE. Women who delivered by elective CS (category 4) [9] were included if they had one or more additional risk factors, whereas all women who delivered by emergency CS (category 1, 2, or 3) [9] were included in the study [6]. Other inclusion criteria included the absence of active bleeding and haemodynamic stability (pulse <100 beats per min and systolic blood pressure >100 mmHg). All participants were deemed capable of providing informed consent. Postpartum haemorrhage (PPH) and severe preeclampsia (PE) were two risk factors for VTE. LMWH was indicated for patients with PPH or severe PE after stabilization of the condition. Women already taking an anticoagulant or having any contraindication to LMWH, such as antenatal or postpartum active bleeding requiring blood transfusion, placenta previa, thrombocytopenia (platelet count <75 × 10^8^/μl), severe renal disease (glomerular filtration rate <30 ml/minute), severe liver disease, or uncontrolled hypertension (>200/120 mmHg), were excluded.

Women who were eligible for participation were assigned to the three arms of the trial: the bemiparin, enoxaparin, and control groups. Based on a schema produced by a Microsoft Excel (2007) computer program, the first woman was recruited to the no-intervention group (control), the second to the bemiparin group, and the third to the enoxaparin group, with this sequence repeated throughout the trial. This method of recruitment was necessary owing to the fact that we were unable to formulate a complete list of all eligible women at one time and recruit them into the three study groups. This is because women who were included in the trial had not been monitored at our centre during pregnancy, and were seen for the first time during labour.

Data were collected by senior house officers and by obstetricians on call in the labour room or when preparing women for elective or emergency CS. These data collectors were trained by the study investigators regarding risk factors for VTE and consent procedures.

Dehydration in all three groups was avoided by administering oral and intravenous fluids during labour and postpartum according to hospital regulations. All women were encouraged to mobilize during labour and the early postpartum period. This is because there were no other types of mechanical methods of thromboprophylaxis present in the hospital.

Interventional drugs (3500 IU bemiparin or 4000 IU enoxaparin) were supplied gratis by the hospital, along with antibiotics, analgesics, and intravenous fluids. Women were kept in the hospital until they were completely ambulant. After discharge from the hospital, the remaining doses of LMWHs were purchased by the women, together with antibiotics, analgesics, and tonics that are routinely prescribed to women after delivery. All in-hospital services (including CS) and drugs were provided for free during the hospital stay because the Maternity Teaching Hospital is a public hospital and is supported by the government. The costs of bemiparin and enoxaparin were nearly equal and did not constitute a financial burden after discharge because all of the participants were asked regarding their compliance with the use of injections.

Written consent was obtained by doctors attending the delivery room who were all trained by the research team on obtaining informed consent from the participants. Informed consent was obtained following explanation to and discussion with each participant, and after answering questions and queries raised by the participants. Consent was formally documented in the patients’ medical records, and approved by the ethics committee of the Maternity Teaching Hospital.

The trial protocol was approved by the Research Ethics Committee of Hawler Medical University. The same committee acts as an Institutional Review Board committee in our institution and approval was gained from both of them (document No. 1/7, 16/4/2012). The data safety and monitoring committee of the Maternity Teaching Hospital consisting of three independent obstetricians from the hospital ensured continued safety of the patients and ongoing monitoring of adverse events, which were recorded throughout the trial.

Sample size was estimated using the PS Power and Sample Size Calculator, Version 3.0. Information entered into the program included an alpha error of 0.01, a power of 90%, an estimated incidence of VTE among women who did not receive thromboprophylactic LMWH of 2 per 1000 [10,11], and an estimated incidence of VTE among women who received enoxaparin of 1.15% [12]. The estimated sample size was 2209 per group. To the best of our knowledge, no study has estimated the incidence of VTE in women taking bemiparin. Therefore, the estimated sample size was the same for bemiparin as for enoxaparin. To allow for patients’ loss to follow-up, each study group consisted of 2340 women.

Women who were recruited to the enoxaparin group received injections of pre-filled syringes of enoxaparin sodium 40 mg (Clexane Sanofi-Aventis; equivalent to 4000 IU anti-Xa activity) in 0.4 mL water. Women who were recruited to the bemiparin group received injections of 3500 IU bemiparin (Hibor, Laboratories Fcos ROVI, SA, Madrid, Spain) in 0.2 mL water.

The first dose of bemiparin (3500 IU/) or enoxaparin (4000 IU) was injected subcutaneously into the upper aspect of the arm, around the umbilicus, or in the upper aspect of the thigh 6 hours after vaginal delivery or CS under general anaesthesia. In women who were administered spinal anaesthesia, the first dose was administered 8 hours after delivery. The time of receiving the first dose was recorded in the files of the patient. The second dose (3500 IU bemiparin or 4000 IU enoxaparin) was delivered 24 hours later, and then daily up to a total of seven doses. Women with severe PE or PPH received the first dose of LMWH 8–24 hours after delivery.

Stable patients were discharged from the hospital on the 3rd day after CS when they were completely ambulant and 24 hours after vaginal delivery. Women were examined on the 7th day postpartum and 6 weeks after delivery, and were regarded as free of VTE if there was no sign of symptomatic VTE 40 days postpartum. Participants confirmed regular intake of the interventional drugs when they were asked about this issue 7 days post-delivery. All of the women were informed regarding the clinical features of VTE. Signs and symptoms of deep vein thrombosis (DVT) were defined for the participant as the feeling of pain, swelling, tenderness, discoloration, or redness of the affected area, and skin that is warm to the touch [13]. Signs and symptoms of PE were defined as the development of shortness of breath, rapid heartbeat, sweating and sharp chest pain [14]. Women were asked to attend the hospital if they experienced any of these symptoms.

Women presenting with clinical signs or symptoms of DVT were admitted to the hospital. DVT was confirmed by compression ultrasound or magnetic resonance imaging. Women presenting with signs and symptoms of PE were referred to the intensive care unit of Erbil Teaching Hospital. PE was confirmed by computed tomography pulmonary angiography, and women were admitted to the unit and remained under observation until they were stable and discharged home.

The primary outcome measure was the incidence of symptomatic VTE in the three groups. Secondary outcome measures included the incidence of side effects and wound complications in the two intervention groups. Side effects included bruising or pain at the site of injection, ecchymosis, allergic skin reactions, itching, urticaria, and wound haematoma, separation, or dehiscence.

Data were analyzed using the Statistical Package for Social Sciences (SPSS, version 19). Proportions were compared using the chi square test of association, or when applicable, Fisher’s exact test. Binary logistic regression analysis was used with VTE as the dependent variable. Variables showing significant association with VTE (by chi square tests) were entered into a logistic regression model as independent variables. A P value ≤ 0.05 was considered statistically significant.

## Results

**Figure 1:**
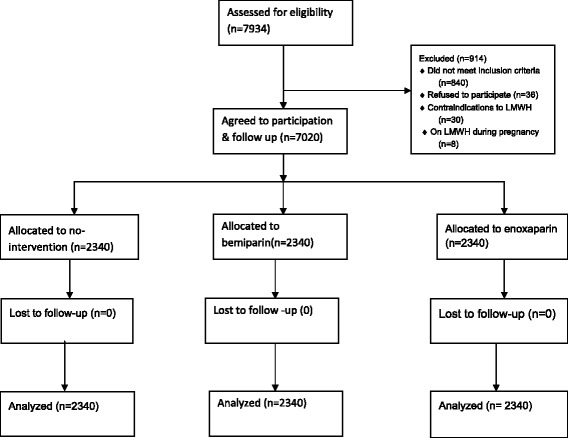
**Flowchart of the trial.**

Figure 1 shows the trial profile. Of the 7934 women interviewed for eligibility, 840 did not meet the inclusion criteria,36 refused to participate, eight were on an anticoagulant during pregnancy, and 30 had contraindications to heparin (including active bleeding in 11, nine had thrombocytopenia, and 10 had severe hypertension). Therefore, 7020 women were recruited and assigned at a ratio of 1:1:1 to the three study groups (n = 2340 per group). No women dropped out prior to assessment of outcome at 40 days in any of the three groups because the researchers only involved women who were interested in being followed up for 40 days postpartum and were on medication as a thromboprophylaxis.

The baseline demographic and clinical characteristics of the three groups, including the prevalence of risk factors for VTE, are shown in Table 1.

**Table 1:** **Baseline demographic and clinical characteristics, including the prevalence of risk factors for VTE**

**Risk factors for VTE**	**Enoxaparin**		**Bemiparin**		**Control**	
	**n = 2340**		**n = 2340**		**n = 2340**	
	**No.**	**%**	**No.**	**%**	**No.**	**%**
-History of APS	0	0	2	0.1	0	0
-Varicose veins	33	1.4	31	1.3	31	1.3
-Heart disease	5	0.2	7	0.3	7	0.3
-Surgery during pregnancy	32	1.4	26	1.1	26	1.1
-Dehydration	10	0.4	18	0.8	2	0.1
-Severe inflammation	15	0.6	16	0.7	0	0
-Pre-eclampsia	147	6.3	123	5.3	125	5.3
-Prolonged labour	173	7.4	148	6.3	174	7.4
-Smoking	42	1.8	31	1.3	36	1.5
-Twin pregnancy	70	3.0	54	2.3	57	2.4
-Postpartum haemorrhage	23	1.0	24	1.0	22	0.9
-Bed rest >3 days	4	0.2	24	1.0	4	0.2
-Wound infection	7	0.3	22	0.9	7	0.3
-Pyelonephritis	8	0.3	37	1.6	28	1.2
-BMI > 30 kg/m^2^	1222	52.2	1335	57.1	1197	51.2
-Age > 35 years	852	36.4	938	40.1	864	36.9
-Para ≥ 4	1201	51.3	1133	48.4	1156	49.4

VTE:venous thromboembolic event; APS: antiphospholipid antibody syndrome; BMI: body mass index.

The primary outcome, symptomatic VTE, was observed in one (0.043%) woman in the bemiparin group, two (0.085%) in the enoxaparin group, and nine (0.384%) in the control group (P = 0.017, Table 2) for the three modes of delivery. The incidence of symptomatic VTE was 0.5% in women with a body mass index (BMI) < 25 kg/m^2^,0% in women with a BMI of 25–30 kg/m^2^, and 0.2% in women with a BMI >30 kg/m^2^ (P = 0.003). No other factor was significantly associated with the incidence of VTE. All cases of VTE occurred within the first week after delivery.

**Table 2:** **Relationship between risk factors and the incidence of VTE**

**Variables**	**N**	**Venous thromboembolism**		**P**
		**No.**	**%**	
Interventional groups				
Control	2340	9	0.384	0.017*
Bemiparin	2340	1	0.042	
Enoxaparin	2340	2	0.085	
Age (years)				
≤35	4366	8	0.18	1*
>35	2654	4	0.15	
BMI (Kg/m^2^)				
<25	586	3	0.5	0.003*
25-29	2680	0	0	
30+	3754	9	0.2	
Risk of APS				
No	7018	12	0.2	1*
Yes	2	0	0	
Varicose veins				
No	6925	12	0.2	1*
Yes	95	0	0	
Heart Disease				
No	7001	12	0.2	1*
Yes	19	0	0	
Surgery during pregnancy				
No	6936	12	0.2	1*
Yes	84	0	0	
Dehydration				
No	6990	12	0.2	1*
Yes	30	0	0	
Severe inflammation				
No	6989	12	0.2	1*
Yes	31	0	0	
Pre-eclampsia				
No	6625	10	0.2	0.144*
Yes	395	2	0.5	
Prolonged labour				
No	6525	10	0.15	0.206*
Yes	495	2	0.4	
Smoking				
No	6911	12	0.17	1*
Yes	109	0	0	
Twin pregnancy				
No	6839	11	0.16	0.269*
Yes	181	1	0.55	
PPH				
No	6951	12	0.17	1*
Yes	69	0	0	
Bed rest				
No	6988	12	0.17	1*
Yes	32	0	0	
Wound infection				
No	6984	12	0.17	1*
Yes	36	0	0	
Pyelonephritis				
No	6947	12	0.17	1*
Yes	73	0	0	
IVF				

*Analysed by Fisher’s exact test.

BMI: body mass index; APS:antiphospholipid antibody syndrome; PPH: postpartum haemorrhage; IVF: *in vitro* fertilisation.

Women on bemiparin were at lower risk of developing symptomatic VTE than women in the control group (odds ratio [OR] =0.106; 95% confidence interval [CI] = 0.013–0.838, Table 3). However, BMI was not significantly associated with the incidence of VTE.

**Table 3:** **Factors significantly associated with VTE by logistic regression**

**Variables**	**B**	**P**	**OR**	**95% C.I. for OR**	
				**Lower**	**Upper**
LMWH		.028			
Control (Reference)			1		
Bemiparin	−2.245	.033	.106	.013	.838
Enoxaparin	−1.486	.058	.226	.049	1.049
BMI categories (kg/m^2^)		.615			
<25 (Reference)			1		
25-29	−15.811	.983	.000	.000	
30+	-.660	.324	.517	.139	1.920
Constant	−4.525	.000	.011		

LMWH: Low-molecular-weight heparin; BMI: body mass index; B: regression coefficient; OR: odds ratio; CI: confidence interval.

Table 4 shows that the proportion of women experiencing mild side effects (pain and ecchymosis) was significantly lower in the bemiparin group than in the enoxaparin group. Wound dehiscence, hematoma, and separation were observed in six women in the enoxaparin group, but in no women in the bemiparin group (P = 0.031).

**Table 4:** **Side effects and complications of enoxaparin and bemiparin**

**Side effects and complications**	**Enoxaparin**		**Bemiparin**		**P**
	**No. (n = 2340)**	**%**	**No. (n = 2340)**	**%**	
Pain	45	1.9	20	0.85	0.002
Ecchymosis	33	1.4	21	0.89	0.1
Wound dehiscence	6	0.256	0	0	0.031*
Wound separation	6	0.256	0	0	0.031*
Wound Haematoma	6	0.256	0	0	0.031*
(Urticaria, Allergy, Itching)	0	0	0	0	NA

*Analysed by Fisher’s exact test.

Six women developed all of the wound complications together. The wounds were separated in one of the ends and there was dehiscence in the other end of the wound. The base of the wounds contained clots and haematomas and there was no sign of infection in the wounds. Wound complications occurred within the first 3 days after receiving enoxaparin. Eighteen women in the control group developed wound infection leading to separation of the edges at 5–10 days post caesarean section.

The incidence of symptomatic VTE was significantly lower in the two combined intervention groups (0.64 per 1000 deliveries) than in the control group (3.8 per 1000 deliveries) (relative risk = 0.166; 95% CI = 0.045–0.614; P = 0.004 by Fisher’s exact test).

Thirty women presented with symptoms suspected to be VTE (in all the 3 groups) all were admitted to the hospital and assessed accordingly. VTE was not diagnosed in these patients, and all were reassured and discharged home. Confirmed cases of DVT were admitted to the hospital. Therapeutic doses of LMWH were prescribed for them and they were discharged home after 48 hours. These women were advised to use the medications at home. Women were followed up for 6 weeks postpartum. Women who were suspected to have PE were transferred to the intensive care unit at Hawler Teaching Hospital where facilities were available to confirm diagnosis, in addition to treatment and follow-up of patients.

One woman died during the study period. She had a twin pregnancy and underwent emergency CS owing to fetal distress. She developed severe dyspnea and cyanosis 5 hours after delivery (1 hour before administration of LMWH). She was in the bemiparin group and died within 10 minutes of resuscitation owing to a major embolus in the pulmonary system. This patient was excluded from the analysis.

## Discussion

Guidelines of the RCOG and the National Institute for Health and Clinical Excellence encourage the use of LMWH as thromboprophylaxis in high-risk pregnancies and during the postpartum period. However, these recommendations were largely based on expert opinion with little evidence from randomized controlled trials and meta-analyses [3].

We found that the combination of the two groups who received either enoxaparin or bemiparin resulted in a postpartum symptomatic VTE rate of 0.64 per 1000 deliveries compared with 3.8 per 1000 in the control group. Both LMWHs were efficient in decreasing the rate of VTE, although enoxaparin was more likely than bemiparin to cause wound complications.

A retrospective cohort study of 653 at-risk gravid women who received guideline-recommended enoxaparin for caesarean thromboprophylaxis showed that 55 (8.5%) of these women experienced wound complications, including separation and/or hematoma [15]. The factor Xa-IIa ratio is higher for bemiparin than for enoxaparin, which may explain the higher rates of hematoma and other complications in the enoxaparin group [8].

Operative delivery has been reported to place women at risk for VTE [5,16-18]. Most studies on thromboprophylaxis involved women who delivered via CS [19,20]. A previous study assessed thromboprophylaxis with unfractionated heparin in 116 parturients with varicose veins who delivered vaginally or by CS, and showed that unfractionated heparin significantly reduced the incidence of postpartum venous thrombosis [21]. Notably, we found that vaginal delivery also entailed a risk of developing VTE in the absence of thromboprophylaxis. Of the780 women in the control group who delivered vaginally, two (0.256%) developed symptomatic VTE compared with no women who delivered vaginally and were administered bemiparin or enoxaparin. The rate of VTE in previous studies ranged from 1–3 per 1000 deliveries [22-25]. We observed a rate of incidence of VTE of 3.8 per 1000 deliveries. The higher rate observed in our study was likely due to inclusion of women at risk for VTE, whereas previous studies assessed the rate of postpartum VTE, irrespective of risk factors or the mode of delivery.

The major strength of this study was that it was performed in a general public hospital, with over 24,000 deliveries per year. This allowed a relatively large sample size to accrue within a relatively short period of time. The Maternity Teaching Hospital is the only public hospital in Erbil City and is regarded as a tertiary centre.

One limitation of this study was its open-label design. We found it difficult to provide the same-shaped pre-filled syringe containing different materials. The study was not funded by any drug company, making it difficult to provide placebo injections. Another limitation was the absence of lists of women needing thromboprophylaxis. Therefore, a pre-prepared method of allocation concealment could not be formulated for the purpose of designing the trial as a randomized clinical trial. Additionally, the women had to purchase the two interventional drugs after discharge from the hospital. There were no regulations and guidelines for postpartum thromboprophylaxis at the beginning of the research. However, at the end of this study, most of the obstetricians practicing at the Maternity Teaching Hospital became familiar with the possibility of VTE and its danger to patients, thus becoming aware of its signs and symptoms and modes of prevention. This encouraged the development of local guidelines for thromboprophylaxis and treatment of VTE during pregnancy and the postpartum period.

Additional research is required to determine the optimal time for administering LMWH. The death of one woman suggests that administering thromboprophylaxis earlier than 6 hours may prevent formation of thrombus. Additional randomized double-blind trials are required to mask the interventional drugs from the investigators.

## Conclusions

Postpartum bemiparin and enoxaparin are both effective as prophylaxis for VTE. Wound complications develop after enoxaparin, but not after bemiparin use.
